# Donor genetics and storage conditions influence mitochondrial DNA and extracellular vesicle levels in RBC units

**DOI:** 10.1172/jci.insight.187792

**Published:** 2025-06-10

**Authors:** Xutao Deng, Clara Di Germanio, Erika G. Marques de Menezes, Pamela Milani, Mars Stone, Heather Tanner, Sonia Coco Bakkour, Daniel M. Chafets, Sarah E. Reese, Nareg H. Roubinian, Steven Kleinman, Tamir Kanias, Michael P. Busch, Eric J. Earley, Grier P. Page, Travis Nemkov, Angelo D’Alessandro, Philip J. Norris, for the Recipient Epidemiology and Donor Evaluation Study-IV-Pediatric (REDS-IV-P)

**Affiliations:** 1 Vitalant Research Institute, San Francisco, California, USA.; 2 UCSF, San Francisco, California, USA.; 3 Westat, Rockville, Maryland, USA.; 4 Kaiser Permanente Northern California Division of Research, Pleasanton, California, USA.; 5 University of British Columbia, Victoria, British Columbia, Canada.; 6 University of Colorado, Denver-Anschutz Medical Campus, Aurora, Colorado, USA.; 7 Research Triangle Institute International, Atlanta, Georgia, USA.; 8 Omix Technologies Inc., Aurora, Colorado, USA.; 9 See Supplemental Acknowledgments for details on the REDS-IV-P.

**Keywords:** Cell biology, Hematology, Innate immunity

## Abstract

Extracellular vesicles (EVs) and mtDNA have been found in blood products and can activate immune cells; we sought to characterize their evolution in stored blood products. From a previous study of hemolysis in 13,403 blood donors, a second blood unit was drawn from 651 donors and sampled at days 10, 21, and 42. EV counts and RBC-EVs increased with storage time, and EV levels were higher in males and in RBC units processed in AS-1 compared with AS-3. mtDNA levels were higher in females and RBC units processed in AS-3. EV populations and mtDNA levels were highly correlated within donors for 98 donations obtained 2–12 months apart. Quantitative trait locus analysis revealed several genetic associations, most notably linking mtDNA levels with polymorphisms in *ANKLE1*, which encodes an erythroid-specific protein that preferentially cleaves mtDNA. These data suggest that donor-intrinsic factors may influence mtDNA and EV levels found in RBC units. This finding lends impetus to determining if genetic or environmental factors control levels of these immune mediators in blood donors.

## Abstract 2

We found that extracellular vesicle and mitochondrial DNA levels are stable within blood donors over time and discovered genetic linkage for mitochondrial DNA levels.

## Graphical abstract

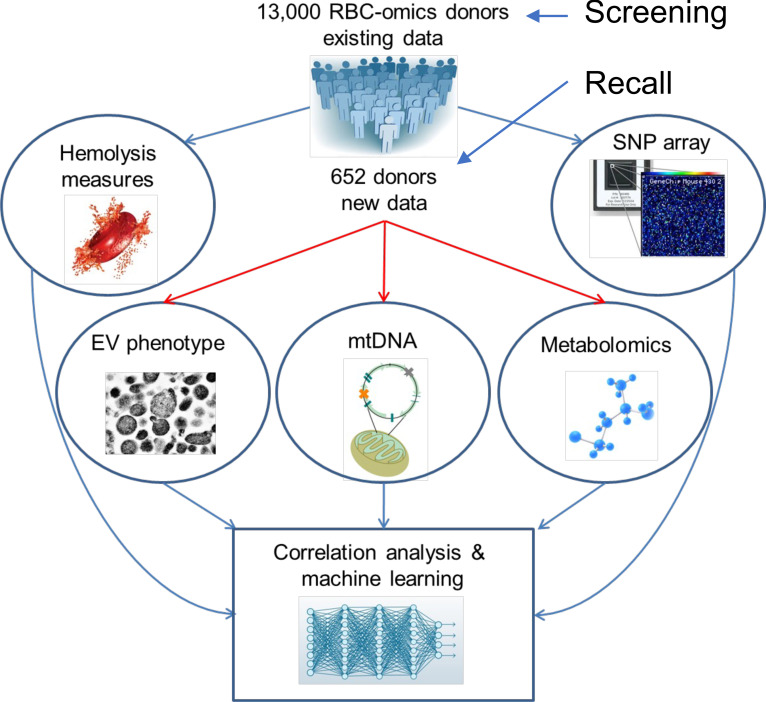

## Introduction

Storage of RBCs allows widespread access to life-saving transfusions. However, transfusion of blood at the end of storage causes transient increased bilirubin and serum iron in transfusion recipients (1). Large randomized clinical trials did not show a difference in mortality or change in multiple organ dysfunction score (MODS) in recipients of RBCs stored a median 6–7 versus 22–28 days (2, 3). These results were in contrast to prior animal model data, including a mouse model showing increased immune activation after receipt of stored but not fresh RBCs (4) and a guinea pig model showing aged RBC infusion causes damage to the vascular walls and kidneys (5). Given the design of the clinical trials with a small minority of transfused RBC units stored > 35 days, it is still not clear if RBC units transfused at the end of storage (36–42 days) have reduced efficacy or harmful effects (6). Therefore, understanding how the characteristics of RBCs change during storage remains an important area of research.

During RBC storage, extracellular vesicles (EVs) are generated by RBCs, and their characteristics can change with storage time (7). Elevated levels of EVs in stored RBCs have been associated with in vitro procoagulant activity (8), activation of lung cancer cells in vitro (9), and increased endothelial cell permeability (10). Similarly, mitochondrial DNA (mtDNA) can be released from cells into the blood and can bind to TLR9 expressed on RBCs and drive innate immune activation (11). High levels of mtDNA in stored RBCs have been linked to development of adult respiratory distress syndrome (ARDS) in transfused trauma patients (12). Understanding and monitoring EVs and mtDNA during RBC storage could enhance the quality and safety of transfusion practices.

During storage, RBCs undergo metabolic shifts leading to accumulation of byproducts like lactate and potassium, depletion of ATP, and oxidative damage (13). These changes, collectively known as the “storage lesion,” can compromise the quality of stored RBCs and, potentially, their therapeutic effectiveness. Metabolomics can identify biomarkers of storage-induced alterations and develop strategies to mitigate these effects, such as optimizing storage conditions or supplementing storage solutions with metabolic substrates (14).

The current manuscript builds on a genome-wide association study (GWAS) performed using samples from 13,403 donors enrolled into the RBC-Omics protocol of the Recipient Epidemiology and Donor Evaluation Study (REDS-III). Recall of 651 of the RBC-Omics donors who exhibited the extremes of high or low hemolysis (*n* = 502) or were high-intensity donors (≥9 RBC donations over 24 months, *n* = 150) revealed that osmotic and oxidative hemolysis were highly donor dependent and consistent across time (15). This implied the potential for genetic control of osmotic and oxidative hemolysis of RBCs, and the GWAS revealed several potentially novel associations with these parameters (16). In the current study, serial samples from recalled donors were tested for the potential inflammatory mediators mtDNA and EVs, and an assessment of the metabolomic profile of the RBC units was performed.

## Results

### Dynamics of EVs and mtDNA levels with storage of leukoreduced (LR)-RBC units.

**Figure 1:**
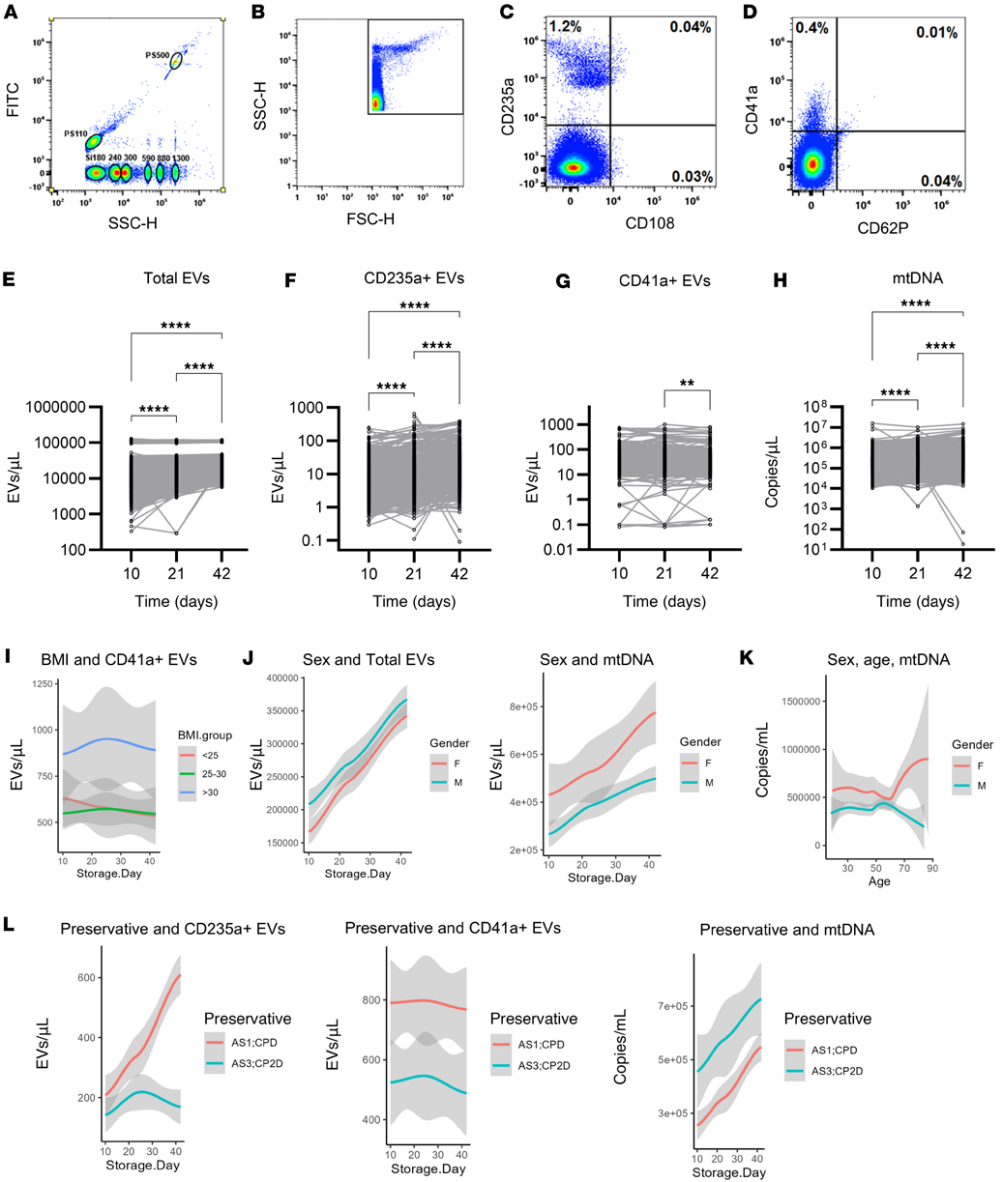
Evolution of EV and mtDNA over RBC unit storage time. (**A**) FITC-labeled polystyrene beads of 110 and 500 nm are shown, as well as silica beads sized 180, 240, 300, 590, 880, and 1,300 nm. (**B**) The forward and side scatter gate for the EV population, approximately corresponding to silica beads from 180 to 1,000 nm on the SSC channel. (**C**) Very few CD235a^+^ RBC EVs express CD108. (**D**) A minority of CD41a^+^ platelet EVs coexpress CD62P. (**E**–**H**) Samples collected from 651 RBC units at days 10, 21, and 42 were tested for total EVs (**E**), CD235^+^ EVs (**F**), and CD41a^+^ EVs (**G**), as well as mtDNA (**H**). For EV measurements, 634, 641, and 647 samples were tested for the day 10, 21, and 42 time points, respectively. For mtDNA measurement, 639, 630, and 646 samples were tested for the day 10, 21, and 42 time points, respectively. Comparison across time points was made by 1-way ANOVA with a Holm-Šidák post hoc test. **P < 0.01, ****P < 0.0001. Linear mixed models were generated to test whether donor age, sex, BMI, or race or RBC preservative solution was associated with EV or mtDNA levels. The lines in **I**–**L** represent the conditional mean calculated by LOESS regression, and the shaded areas represent the 95% CI. (**I** and **J**) Donor BMI was associated with CD41a^+^ EVs (p = 0.019) (**I**), donor sex was associated with total EV level (*P* = 0.032) and mtDNA level (*P* = 0.002) (**J**). (**K**) Examination of mtDNA level by donor age showed larger differences between males and females after age 60. (**L**) preservative solution was associated with CD235a^+^ EVs (*P* < 0.0001), CD41a^+^ EVs (*P* = 0.006), and mtDNA (*P* = 0.002).

Supernatant samples from RBC storage bags were acquired at days 10, 21, and 42 of storage. Samples were stored in AS-1 (*n* = 188) or AS-3 (*n* = 464), depending on the collecting blood center standard practice. EVs were gated to enumerate CD41a^+^ EVs (platelet origin) and CD235a^+^ EVs (RBC origin), (Figure 1, A–D). Only low numbers of platelet or RBC EVs coexpressed CD62P or CD108, respectively. Given the very low levels of CD108 and CD62P expression measured on EVs, these data were not further analyzed. The range of EV concentrations spanned 2–3 logs among donors for both RBC and platelet-derived EVs (Figure 1, E–G). Total EV counts rose during storage, consistent with the increase in RBC-derived EVs. Platelet-derived EVs decreased from day 21 to day 42, consistent with our prior observations (17). Levels of mtDNA showed a similar 3-log variation among donors (Figure 1H), and while levels rose modestly through storage time, the changes over time were smaller than the variation from donor to donor, also consistent with prior work (18). Total EV levels did not show a significant correlation with mtDNA (*P* = 0.5), though platelet EVs showed a negative correlation (*r* = –0.19, *P* < 0.001) and RBC EVs showed a positive correlation (*r* = 0.46, *P* < 0.001) with mtDNA levels (Supplemental Figure 1A; supplemental material available online with this article; https://doi.org/10.1172/jci.insight.187792DS1). To determine the proportion of mtDNA that is contained in EVs, supernatant samples were treated with Triton, DNase, or Triton plus DNase. DNase alone should only degrade mtDNA not protected by EVs, while Triton plus DNase should degrade free and EV-protected mtDNA. The mean signal for mtDNA fell by 1.7 Cq or 3.2-fold with addition of DNase (*P* = 0.36) and by 6.0 Cq, or 64-fold with addition of Triton plus DNase (*P* < 0.001; Supplemental Figure 1B). These results are consistent with 85% of mtDNA residing in EVs in these samples.

### Effect of blood processing and donor characteristics on RBC unit EV and mtDNA levels.

Variation between donors was seen in EV and mtDNA levels across all 3 time points studied, and we explored whether RBC unit processing or donor characteristics were associated with these parameters. Donor race and age were not found to be associated with EV or mtDNA levels. Donor BMI > 30 kg/m^2^ (clinically defined as obese) was associated with higher levels of platelet-derived EVs (*P* = 0.019; Figure 1I), and male sex was associated with higher levels of total EVs (*P* = 0.032) and lower levels of mtDNA (*P* = 0.002) across storage time (Figure 1J). Notably, the level of mtDNA rose sharply with age in postmenopausal-aged females (Figure 1K). Finally, storage in AS-1 compared with AS-3 was associated with higher levels of RBC EVs (*P* < 0.0001) and platelet EVs (*P* = 0.006) and with lower levels of mtDNA (*P* = 0.002; Figure 1L). Three of the 4 participating blood centers performed immediate leukoreduced (LR), while the fourth delayed LR 48–72 hours after collection. Delayed LR caused increased RBC and platelet EV but not total EV or mtDNA levels across storage time points (Supplemental Figure 2).

### Relationship between EV and mtDNA levels with RBC unit metabolites.

**Figure 2:**
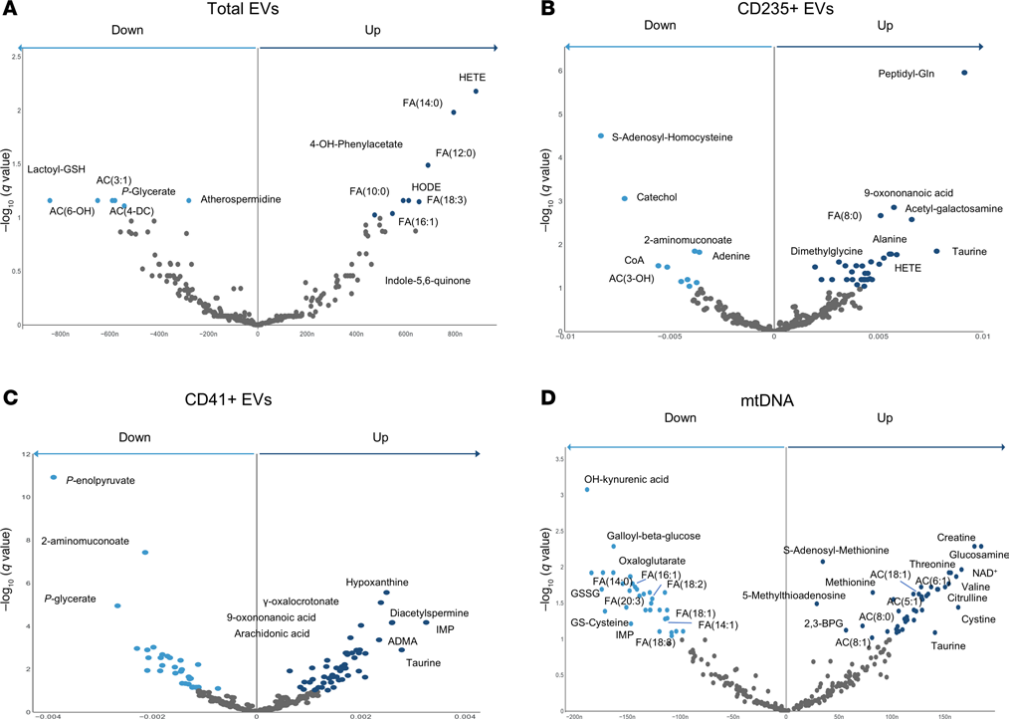
Metabolite correlations with EV populations and mtDNA. Volcano plots indicate metabolite associations to EV populations or mtDNA (*n* = 651), with the *x* axis indicating the Spearman-determined correlation between hemolysis and metabolite levels and the *y* axis indicating the –log_10_ of the FDR-corrected *P* values for such associations. Significant negative associations (*q* < 0.1) are shown in light blue, and significant positive correlations are shown in dark blue. Selected significant metabolites are labeled. AC, acyl carnitine; FA, fatty acid; HODE, hydroxyoctadecadienoic acid; HETE, hydroxyeicosatetraenoic acid; IMP, inosine monophosphate; ADMA, asymmetric dimethylarginine; GSSG, glutathione disulfide.

We next tested whether the EV phenotype or mtDNA content in the LR-RBC unit would be correlated with RBC metabolite levels. Consistent with hemolysis correlation results, these analyses revealed several subtle but significant correlations. Top positive correlates with total EV count included numerous free fatty acids (FA) such as FA(14:0), FA(12:0), FA(10:0), FA(18:3), and FA(16:1) as well as the pools of hydroxyeicosatetraenoic (HETE) and hydroxyoctadeadienoic (HODE) acids. Conversely, the methylglyoxal pathway intermediate, lactoyl-glutathione (GSH), late glycolytic intermediate, phosphoglycerate, and short chain acylcarnitines (AC) AC(3:1), AC(4-DC), and AC(6-OH) were inversely correlated with total EVs (Figure 2A). Specific analysis of HETEs (19) revealed that the top correlates with total EV count were 15(S)-HETE, 5(S)-HETE, and 12(S)-HETE (Supplemental Table 2). Likewise, HETE and FA(8:0) positively correlated with RBC-derived EVs, along with alanine, taurine, and dimethylglycine (Figure 2B). Platelet EVs also positively correlated with taurine, FA(20:4) (arachidonic acid), and the purine catabolite hypoxanthine (Figure 2C), the latter of which is a notable correlate with posttransfusion recovery (20, 21). Finally, mtDNA abundance positively correlated with various AC including AC(6:1), AC(5:1), AC(18:1), AC(8:0), and AC(8:1) and inversely correlated with various FA including FA(14:0), FA(16:1), FA(18:2), FA(18:1), FA(18:3), and FA(14:1) (Figure 2D). In addition, positive correlates were observed for mtDNA levels with methionine, S-adenosyl-methionine, and 5-methylthioadenosine (Figure 2D).

### Correlation of EV and mtDNA levels with hemolysis in RBC units.

We next tested whether EV phenotype or mtDNA content in the LRpRBC unit would be correlated with RBC hemolysis measures. While several of the correlations were significant, in general, the correlation coefficients between the measured parameters and each of the 3 RBC hemolysis measures were low (Spearman ρ < 0.2). The parameters showing the strongest correlations with storage hemolysis were total EV concentration at days 10 and 21 and RBC-derived EV concentration at day 42 (Table 1 and Supplemental Figure 3A). Raw data for selected correlations are shown (Supplemental Figure 3, B and C). The only significant association for mtDNA was an inverse correlation with osmotic hemolysis, which was independent of RBC unit storage duration (Supplemental Figure 4A). Finally, we tested whether or not hemolysis parameters were associated with a storage solution and found that storage hemolysis was lower in RBC units stored in AS-3 compared with AS-1 (mean 0.44% versus 0.50%, *P* = 0.031; Supplemental Figure 4, B–D).

### Correlation of EV and mtDNA levels with clinical outcomes in transfusion recipients.

We next tested whether the EV or mtDNA content in the donor unit would be correlated with clinical outcomes in transfusion recipients. The screening units were eligible for transfusion to recipients, since only a small transfer bag was removed from each unit at the start of storage. Of the 651 donors whose EVs and mtDNA were measured in the recall unit in this study, 302 had a unit of LR-pRBCs linked to 487 transfusion recipients (Supplemental Table 1) who received a single unit of blood, for a total of 496 informative transfusion episodes (donors could donate multiple times during the study period). The storage age of each transfused unit was known, and the corresponding EV or mtDNA levels from the recall unit at the storage time point closest to the transfused unit age was assigned. Recipient parameters that were measured were: change in hemoglobin, creatinine, and bilirubin after transfusion. Clinical events like TRALI were not able to be linked individual transfusions. Less than half the recipients had data for change in bilirubin after transfusion, but the parameter was included in the analysis since a rise in bilirubin could be a marker for intravascular hemolysis. Extreme outliers for each of these parameters were excluded. None of the EV or mtDNA levels correlated with changes in hemoglobin, creatinine, or bilirubin after transfusion (Supplemental Figure 5).

### Predictive value of metabolite, EV, and mtDNA levels for hemolysis measures.

**Figure 3:**
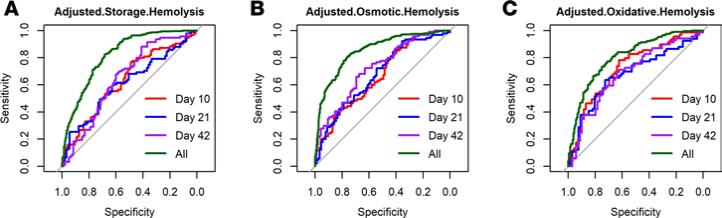
Predictive value of metabolites, EVs, and mtDNA for hemolysis markers. For each hemolysis marker measured, analytes were correlated with hemolysis using machine learning methods, and the results of the support vector machine (SVM) analyses are shown. (**A**–**C**) For each correlation, models were created using the data from storage day 10, 21, or 42 analytes or all storage time points combined (All) to predict day 42 hemolysis for spontaneous hemolysis (**A**), osmotic hemolysis (**B**), and oxidative hemolysis (**C**).

We next questioned whether the parameters we measured would predict hemolysis parameters of RBC units after 42 days’ storage. In preliminary analyses, we tested several machine-learning algorithms, including Caret-PLS, Decision Tree-RPART (Recursive Partitioning for Classification), Random Forest, and support vector machines (SVM). The SVM trained on the training set yielded the highest AUC values, indicating the best predictive value on the test set. Machine-learning analysis of all the metabolites, EV, and mtDNA measures in this study yielded good predictive value for day 42 hemolysis. The composite of analytes at day 10 of storage were relatively poor predictors of day 42 spontaneous hemolysis (AUC = 0.63), but analytes at day 42 of storage correlated well with spontaneous hemolysis (AUC = 0.82; Figure 3A). For day 42 osmotic hemolysis, the composite of analytes at day 10 of storage had similar predictive value, with AUC = 0.67, and analytes at day 42 of storage had better correlation, AUC = 0.85 (Figure 3B). Finally, the best predictive value of machine learning for the available analytes at day 10 was for day 42 oxidative hemolysis (AUC = 0.73), and the correlation with day 42 analytes showed AUC = 0.81 (Figure 3C).

### Stability of EV and mtDNA measurements across repeat donations.

**Figure 4:**
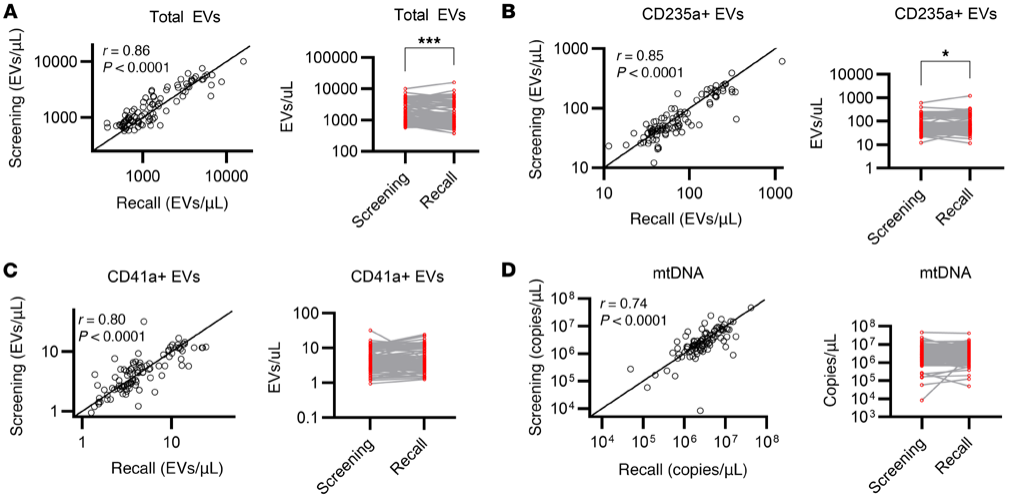
Stability of RBC unit EV and mtDNA measurements across separate blood donations. LR-pRBC were stored for 42 days at 4°C, and aliquots were frozen and stored from the screening and recall visits, which were blood donations separated by 2–12 months (*n* = 98). (**A**–**D**) Paired samples from each participant were each tested in the same batch for total EVs (**A**), CD235a^+^ EVs (**B**), CD41a^+^ EVs (**C**), and mtDNA (**D**). Left panels: The solid line represents the line of identity. Pearson correlation and *P* values for the correlations are shown. Right panels: Paired screening and recall measurements are shown. Populations were compared by Wilcoxon matched-pairs signed rank test. *P < 0.05, ***P < 0.001.

Given the large variation in both EV and mtDNA levels from donor to donor, we queried how stable these parameters were from donation to donation. The screening and recall donations were separated by 2–12 months (15). To test this, we selected 100 donors’ paired samples to compare between the screening and recall donations. Because a supernatant sample was not generated in the screening phase of the study, we used “postfreeze” supernatants to test both donations (Supplemental Figure 6). Surprisingly, total EV counts were relatively stable in samples collected months apart (Figure 4A). Levels of RBC-derived EVs showed slightly tighter correlation compared with platelet-derived EVs (Figure 4, B and C). mtDNA levels also showed strong correlation in samples separated by 2–12 months (Figure 4D). Comparing the median levels in recall versus screening units, total EVs were 1.4% lower, platelet-derived EVs were 13% lower, RBC-derived EVs were 19% higher, and mtDNA was 4.2% higher in recall RBC units (Figure 4).

### Genetic associations with EV and mtDNA levels.

**Figure 5:**
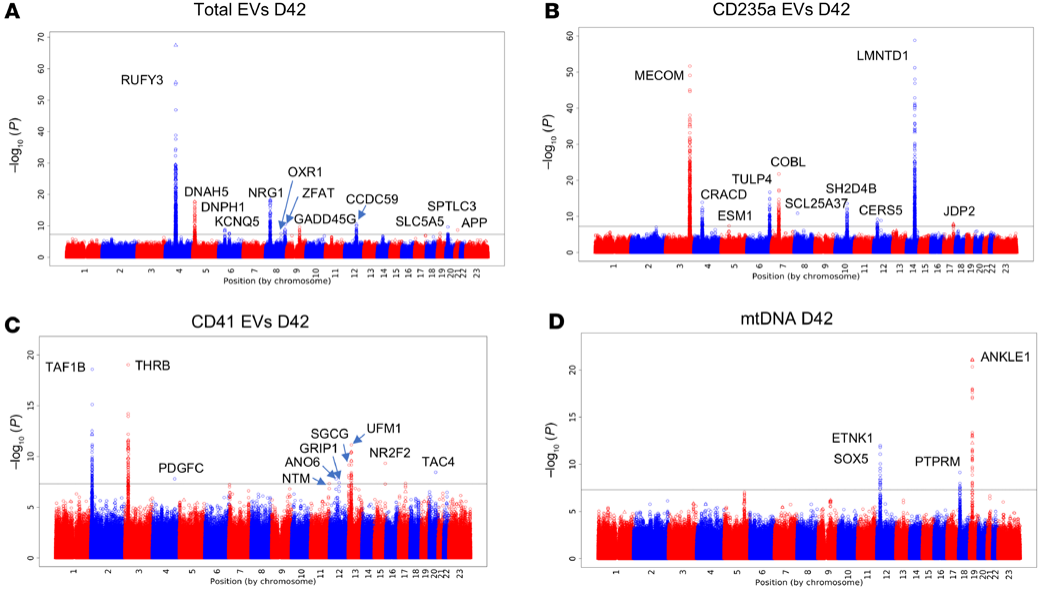
Genetic polymorphisms associated with RBC unit EV and mtDNA levels through storage. (**A**–**D**) A QTL analysis was performed to associate SNPs with total EVs (**A**), CD235^+^ EVs (**B**), and CD41a^+^ EVs (**C**), as well as mtDNA (**D**) in samples collected from 651 RBC units at day 42. Associations with *P* < 5 × 10^–8^ are labeled with the gene name, and the horizontal line on each plot represents *P* = 5 × 10^–8^.

**Figure 6:**
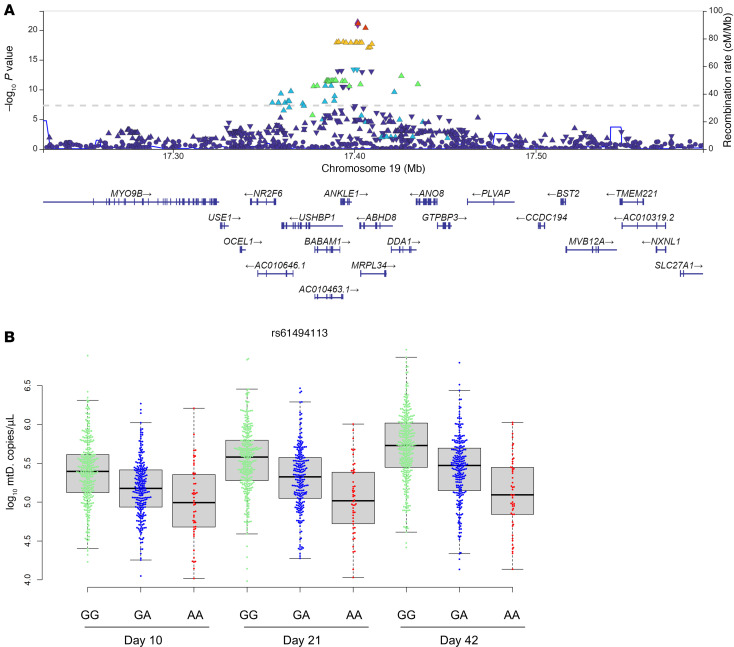
Genetic polymorphisms associated with mtDNA levels through storage. (**A**) LocusZoom plot of the SNPs implicated with day 42 mtDNA levels on chromosome 19. Color coding represents the linkage disequilibrium of each SNP with the lead SNP, rs61494113. Upright triangles represent framestop or splice variations; inverted triangles represent nonsynonymous variations. For each gene shown below the plot, coding regions are represented by vertical bars. (**B**) The association of genotypes for lead SNP rs61494113 with mtDNA levels across storage time.

The relative stability of EV and mtDNA levels in stored blood products collected months apart suggested that there could be a genetic underpinning to the intraindividual correlations. We performed a quantitative trait locus (QTL) analysis using the data from the 651 participants for whom we tested EV and mtDNA levels and metabolomics, performing an independent analysis at each of the storage time points (10, 21, and 42 days). Multiple genetic loci showed variation that reached the genome-wide level of significance for total EV levels or for CD235a^+^ (RBC) or CD41a^+^ (platelet) EVs at day 42 of storage (Figure 5, A–C). While several associations appeared quite strong, such as total EV levels or *RUFY3* for total EV levels and *MECOM* and *LMNTD1* for RBC EVs, none of the associations between SNPs and EV levels were consistent across the 3 storage time points (Supplemental Figure 7, A–C). QTL analysis also revealed several genetic associations with blood product mtDNA levels (Figure 5D). Variations in region of chromosome 19 were significantly associated with mtDNA levels at all 3 storage time points studied (Supplemental Figure 7D), increasing confidence in the association. The LocusZoom plot of the associated SNPs on chromosome 19 reveals several genes in the vicinity of the implicated SNP rs61494113, including *ANKLE1*, *BABAM1*, and *MRPL34* (Figure 6A). The strongest association was with SNP rs61494113, and multiple other SNPs in the region were in linkage disequilibrium with the implicated SNP. The G allele was associated with higher mtDNA levels at all 3 time points studied (Figure 6B).

## Discussion

This study quantified RBC and platelet EVs, as well as mtDNA levels, in LRpRBC units throughout storage, and made a number of observations. RBC unit platelet EV levels were highest in donors with obesity, and males had the highest total EV levels. In contrast, RBC units from postmenopausal females had the highest mtDNA levels. AS-1 compared with AS-3 storage solution was associated with higher RBC and platelet-EV levels and with lower mtDNA levels. Total EV and RBC EV counts correlated with storage hemolysis, while mtDNA levels negatively correlated with day 42 osmotic hemolysis in RBC units. Both EV and mtDNA levels in RBC units were stable within donors across donations separated by 2–12 months. QTL analysis revealed that mtDNA levels at all storage time points were associated with a SNP in chromosome 19 in the vicinity of *ANKLE1*, *BABAM1*, and *MRPL34*. These findings demonstrate that EVs and mtDNA levels in RBC units can be affected by donor intrinsic and extrinsic factors. Better defining determinates of these damage-associated molecular patterns (DAMPs) in blood products is a first step in designing strategies to mitigate potential adverse effects of blood transfusion.

From a metabolomics perspective, total EV counts and RBC-derived vesicles positively correlated with HETE. Generated either enzymatically (lipoxygenase) or nonenzymatically (lipid peroxidation) from linoleic and arachidonic acid, these proinflammatory eicosanoids have been linked to poor RBC storage quality in mice and humans (22, 23). Therefore, these results further substantiate a role for lipid peroxidation in RBC vesicle formation. Of note, the ferrireductase STEAP3 (also known as tumor suppressor-activated pathway 6 [TSAP6]) is a strong regulator of vesiculation (24) and extravascular hemolysis in mice (22). While hits on STEAP3 did not emerge from the QTL analysis for EVs or mtDNA, targeted analysis of STEAP3 SNPs in REDS-III revealed a promising association to these parameters, linking lipid peroxidation to poor hemoglobin increments (25). The positive association between RBC- and platelet EVs with taurine is also notable. Taurine is an abundant, nonessential amino acid that is transported into RBC. Given its antioxidant function specifically in protecting RBC from oxidative damage (26), this positive association suggests a possible adaptive mechanism to cope with a pro-oxidant environment.

Platelet concentrates have been shown to carry mtDNA, (27) with the majority of mtDNA contained in EVs (28). In the current study, mtDNA levels showed an inverse correlation with platelet EVs and a positive correlation with RBC EVs (Supplemental Figure 1A), implying that the latter are more likely important mtDNA carriers in RBC units, though our data also support the majority of mtDNA being carried in EVs. Considering that the units studied were all LR, it is not surprising that residual platelets were not a major source of mtDNA, since LR has been reported to remove 99% of platelets and 72% of platelet EVs (29). Levels of mtDNA strongly correlated with many acylcarnitines and inversely correlated with FAs. These trends are indicative of an activated Lands cycle for membrane lipid repair to cope with oxidative damage. In addition, they positively correlated with methyl-carrying compounds such as methionine, S-adenosyl-methionine, and 5-methylthioadenosine, which are notably involved in methylating oxidatively damaged protein residues as a mechanism of protein repair in RBC (30). The fact that 2 pathways relied upon by RBC for repairing damage are apparently upregulated in RBC units with higher mtDNA suggests higher oxidative environments during erythropoiesis. Healthy individuals’ RBCs lose mitochondria during erythrocyte maturation, though a subset of patients with systemic lupus erythematosus carry RBCs with mitochondria, which is associated with higher disease penetrance (31). RBCs from healthy individuals express TLR9 and can bind mtDNA (32), though the literature is conflicting as to whether mtDNA is preferentially carried in the plasma or bound to RBCs (12, 32, 33).

The most consistent genetic correlation with EVs or mtDNA across storage time points was found between mtDNA and SNPs on chromosome 19. Several genes were potentially associated with the implicated SNP rs61494113, including *ANKLE1*, *BABAM1*, and *MRPL34*. *BABAM1* is regulated by mTORC2 and participates in DNA repair activity (34). *MRPL34* encodes mitochondrial ribosomal protein L34, which assists with protein synthesis in the mitochondrion and would provide a plausible potential indirect link with mtDNA levels in blood products. An even more direct association is between *ANKLE1* and mtDNA, given that its expression is erythroid lineage specific and functions to cleave the mitochondrial genome during erythropoiesis (35). Moreover, ANKLE1 ectopic expression is linked to breast cancer risk (35). SNP rs61494113 represents a G to A variation; the A variant is associated with increased squamous cell cancer risk, with a minor allele frequency of A of 0.3 (36). It has been shown that lower mtDNA in breast cells protects them from apoptosis, which would hypothetically contribute to cancer risk (35), and these findings would be consistent with our study, where the A variant of rs61494113 was associated with lower mtDNA levels. Our findings in RBC units may be clinically important, as the serum level of mtDNA after transfusion is correlated with the transfused dose (12), and mtDNA levels have been associated with risk of lung injury in transfusion recipients (12, 37). The current study showing that mtDNA levels are stable from donation to donation and may be genetically determined opens the possibility to selecting donors whose products are less likely to induce inflammation in transfusion recipients.

This study has some limitations that bear mentioning. The population of 651 donors who were recalled whose RBC units were tested for EV and mtDNA levels may not be representative of the broader donor population, since they were selected based on the extremes of storage, osmotic, and oxidative hemolysis or donation intensity parameters. The fact that both high and low hemolyzing units were included mitigates this limitation, but confirmatory studies should target a broader population. EV and mtDNA measurements were not made on the same unit that was transfused to recipients, and while there was strong correlation of these values across repeat donations, the exact level of these parameters in the transfused unit was not measured. Given that there were only 496 informative transfusion episodes in this study, there was low power to detect adverse clinical events like acute lung injury or hypoxemia after transfusion. Larger, dedicated studies would need to be designed to sufficiently power detection of posttransfusion adverse events.

In summary, detailed study of metabolites, EVs, and mtDNA in aging RBC units revealed how donor intrinsic characteristics and blood storage solution affect these parameters. EV and mtDNA levels were surprisingly stable across donations separated by up to 1 year. Further understanding what regulates both of these potential proinflammatory mediators represents an important area of future research.

## Methods

### Sex as a biological variable.

There were 300 females (95 older than 60) and 352 males (102 older than 60) included in the study. The effect of sex on levels of EVs and mtDNA was analyzed and presented.

### Study participants.

From a parent study of 13,403 blood donors whose RBC units were characterized for spontaneous storage hemolysis, osmotic hemolysis, and oxidative hemolysis, 651 donors were recalled and donated a second RBC unit for longitudinal analysis at days 10, 21, and 42 of storage (15). Donors were selected for the “recall study” if their hemolysis parameters fell within the top or bottom 5% on a given hemolysis measure.

### Sample collections.

The initial study of 13,403 donors (screening phase) collected LR packed RBC (pRBC) units derived from whole blood collection. LR was performed immediately after unit manufacture at 3 of the 4 participating blood centers, while the fourth delayed LR for 48–72 hours after collection (15). An aliquot was removed and stored in a transfer bag, and aliquots were generated after 42 days of RBC unit storage at 4°C. Samples were snap frozen and cryopreserved at –80°C. In addition, a “postfreeze” supernatant was generated to test for mtDNA and EVs in screening samples (Supplemental Figure 5A). In the recall phase of 651 donors, samples were obtained from whole blood LR-pRBC units at days 8–12, 18–23, and 39–42, and the units were not transfused to recipients. At each time point, 15 mL of the LR-RBC component was removed for hemolysis testing, and LR-pRBC and acellular supernatant aliquots were cryopreserved. Repeated parent unit sampling was performed through one of the 2 sampling ports on days 8–12 and 18–23, respectively, using a plasma transfer set (Baxter, 4C2240), while sampling on days 39–42 was performed through the LR-RBC unit main port. For metabolomics testing, the LR-pRBC aliquot was used. For mtDNA and EV testing, the supernatant from the LR-pRBC unit was tested for the 3 time points from the 651 recalled donors, and a “postfreeze” supernatant from the LR-pRBC aliquot was tested for 100 paired samples to compare the day 42 screening and recall phase samples (Supplemental Figure 5B). The 100 paired samples were selected to represent the highest and lowest 16 or 17 samples for each of the 3 hemolysis parameters (spontaneous, osmotic, and oxidative).

### mtDNA quantification.

DNA extraction and real-time PCR were performed as previously described (38). Briefly, frozen RCC supernatants were thawed, and DNA extractions were performed using QIAamp 96 DNA Blood kits (Qiagen). DNA was extracted from 50 μL samples and eluted in 100 μL water. Solution A and B were added to a final concentration of 1×. PCR reactions were performed in triplicate (see Supplemental Methods for details). Serial dilutions of a PCR amplicon containing the target region of mitochondrial cytochrome oxidase III, subunit c, were used to generate a standard curve of spectrophotometer-determined copy number versus Ct. One copy of mtDNA corresponds to 1.8 × 10^–8^ ng based on its length of 16,569 bp. In experiments to measure the proportion of mtDNA contained in vesicles, samples were diluted 1:500 in HBSS (Sigma-Aldrich) containing calcium and split into four 100 μL aliquots. Tubes 2 and 4 had Triton-X100 (Sigma-Aldrich) added (1%), and tubes 3 and 4 had 5 μL NEB DNase I-XT added. After 30 minutes of incubation at 37°C, 100 μL PBS was added to each tube, and the amplification was performed as above.

### EV characterization.

To examine the EVs’ cell of origin and activation markers, 4 fluorochrome-conjugated antibodies were utilized to create a cocktail totaling 6 μL per specimen: RBC markers CD235a-BV421 (clone GA-R2; BD Bioscences, 1 μL) and CD108-PE (clone KS-2; BioLegend, 2 μL) and platelet markers CD41a-PE/Cy7 (clone HIP8; BioLegend, 1 μL) and CD62P-FITC (clone AK4; BioLegend, 2 μL). Prior to testing EV samples, each antibody was titrated to determine the lowest concentration, which yielded nearly maximal fluorescence. Each antibody was filtered using a centrifugal filter (0.22 μm, MilliporeSigma). RBC unit supernatant samples were rapidly thawed; 6 μL of antibody cocktail was added to 10 μL of supernatant and incubated at 4°C for 30 minutes. After staining, samples were diluted 1:100 in buffered 0.22 μm–filtered PBS for immediate flow analysis.

Data were acquired using a 5-laser (UV, violet, blue, yellow/green, and red) Aurora Spectral flow cytometer (Cytek). SpectroFlo QC beads were used to normalize sensor gain. Side scatter was measured using the 405 nm violet laser at a threshold of 1,000 arbitrary units. A 0.22 μm–filtered PBS control was recorded to measure the background signal. Megamix-Plus SSC beads (0.5 mL, Biocytex) and Ultra Rainbow Fluorescent Particles (1 drop, Spherotech) were mixed and acquired to maintain consistent FSC, SSC, and fluorescent voltages as well as EV gates between runs. All samples were acquired at low flow rate (approximately 15 μL/min) and run for exactly 1 minute. EV concentrations were calculated using the flow rate of the cytometer. FCS files were evaluated using SpectroFlo software (Cytek).

### Metabolomics analyses.

Metabolomics extraction and analyses were performed as described (39, 40). RBC samples were transferred on ice to a 96-well plate and frozen at –80°C. Plates were thawed on ice; then, a 10 μL aliquot was transferred with a multichannel pipettor to 96-well extraction plates. A volume of 90 μL of ice-cold 5:3:2 MeOH/MeCN/water (*v/v/v*) was added to each well, with an electronically assisted cycle of sample mixing repeated 3 times. Extracts were transferred to 0.2 μm filter plates (Biotage) and insoluble material was removed under positive pressure using nitrogen. Filtered extracts were transferred to an ultra-high-pressure liquid chromatography system (UHPLC-MS, Vanquish). (UHPLC-MS, Vanquish). Metabolites were resolved on a Phenomenex Kinetex C18 column (2.1 × 30 mm, 1.7 μm) at 45°C using a 1-minute ballistic gradient method in positive and negative ion modes (separate runs) over the scan range of 65–975 *m/z* as previously described (39). The UHPLC was coupled online to a Q Exactive mass spectrometer (Thermo Fisher Scientific). The Q Exactive MS was operated in negative ion mode, scanning in Full MS mode (2 μscans) from 90 to 900 *m/z* at 70,000 resolution, with 4 kV spray voltage, 45 sheath gas, and 15 auxiliary gas. Following data acquisition,.raw files were converted to.mzXML using RawConverter; then, metabolites were assigned and peaks were integrated using ElMaven (Elucidata) in conjunction with an in-house standard library (41).

### Statistics.

Longitudinal changes in EV and mtDNA levels were analyzed by 1-way ANOVA with a Holm-Šidák post hoc test (GraphPad Prism). Associations of EVs and mtDNA with donor characteristics or storage solutions were determined using LOESS regression. The association of EVs and mtDNA with pre-/posttransfusion changes in hemoglobin, total bilirubin, and creatinine after RBC transfusion in recipients was determined using outcomes data from the REDS-III vein-to-vein database using Spearman correlation test (42). An additional filter was applied to the data to exclude observations with outcome values outside of *x* ± 2*s* where *x* is the mean and *s* is the standard deviation of the outcome. Analytes were transformed using log_10_(*x* + 1) where *x* is the analyte. Bivariate associations between each analyte and outcome variables were assessed. All analytes were also assessed in multivariable mixed models for each outcome variable, including those covariates in the Roubinian et al. final models (42). The FDR method was used to adjust for multiple comparisons at *P* < 0.05 (43). All analyses were performed in R, and the mixed-effects linear models were fit using the lme4 R package (44).

To study correlations between EVs, mtDNA, and metabolomics data, hemolysis outcomes were dichotomized into 2 classes (high and low) by their median values. Logarithmic transformation was applied to the predictors of both EV and mtDNA, as well as the metabolomics data. Subsequently, the dataset was divided into 2 random subsets, with 70% allocated to the training set and the remaining 30% to the testing set. The training set was used to train the model, while the testing set was used to evaluate its performance.

A machine learning algorithm was applied to the training set to learn the underlying patterns and relationships in the data. Once the model was trained, it was evaluated on the testing set. In this study, we utilized several widely used algorithms, namely Caret-PLS, Decision Tree-RPART, Random Forest, and SVMs (see Supplemental Methods for further details) (45–48).

A QTL analysis of EV and mtDNA levels was performed as previously described (49). The parent cohort of 13,403 participants was genotyped previously using a Transfusion Medicine microarray consisting of 879,000 single nucleotide polymorphisms (SNPs) (16, 50). After phasing using Shape-IT (51), imputation was performed using Impute2 with the 1000 Genomes Project phase 3 all-ancestry reference haplotypes (52). We used the R package SNPRelate to calculate principal components (PCs) of ancestry (53). We performed association analyses for EVs and mtDNA using an additive SNP model in the R package ProbABE (54). We adjusted for sex, age (continuous), frequency of blood donation in the last 2 years (continuous), blood donor center, and 10 ancestry PCs. Statistical significance was determined using a *P* value threshold of 5 × 10^–8^. We only considered variants with a minimum minor allele frequency of 1% and a minimum imputation quality score of 0.80. The Ensembl Variant Effect Predctor (VEP) was used to annotate the top SNPs (55).

### Study approval.

The study was approved by each site’s IRB, and all participants gave written informed consent.

### Data availability.

Original data are included in the Supporting Data Values file. The metabolomics data associated with this study are deposited in the Metabolic Investigations of Red Blood Cells as a Function of Aging, Genetics, Environment and Storage portal at mirages.shinyapps.io/REDS4_INTERNAL/. Genetic data are available at dbGaP Study (nih.gov) phs001955.v1.p1.

## Author contributions

XD, TN, ADA, PM, and PJN wrote the manuscript. NHR and TK edited the manuscript. DMC, CDG, EGMDM, MS, HT, and SCB and PM oversaw or performed experiments. XD, TN, EJE, GPP, and SER analyzed data. SK, MPB, ADA, and PJN planned the study. REDS-IV-P edited the manuscript.

## Supplementary Material

Supplemental data

ICMJE disclosure forms

Supporting data values

## Figures and Tables

**Table 1:**
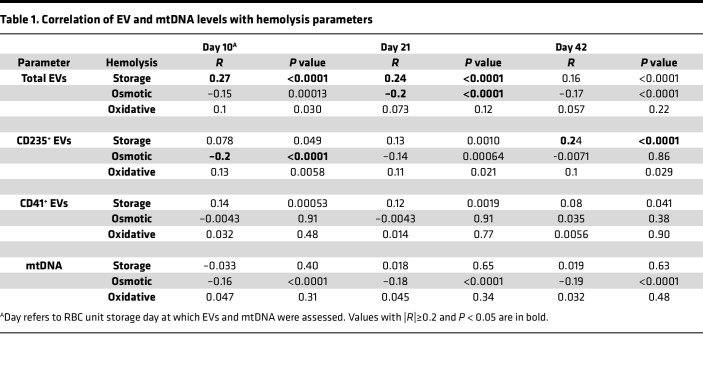
Correlation of EV and mtDNA levels with hemolysis parameters

## Version 1 06/10/2025

In-Press Preview

## Version 2 07/22/2025

Electronic publication
