# Supplementary material for: Donor genetics and storage conditions influence mitochondrial DNA and extracellular vesicle levels in RBC units
Source: JCI Insight. 2025 Jun 10;10(14):e187792. doi: 10.1172/jci.insight.187792 (PMC12288979; doi:10.1172/jci.insight.187792)
Supplement: Supplemental data [file jciinsight-10-187792-s210.pdf]

Methods

MtDNA quantification

MtDNA was amplified using 5 µl of template DNA in a 15 µl reaction containing 5 mM $MgCl_2$, 5 mM dNTPs (Bioline), 1 µM primer CoxF (5’ ATGACCCACCAATCACATGC 3’) and 1 µM primer CoxR (5’ ATCACATGGCTAGGCCGGAG 3’) (Integrated DNA Technologies) specific for mitochondrial cytochrome oxidase III subunit c, 0.25× SYBR Green I (Molecular Probes) and 0.7 U FastStart Taq (Roche Applied Science). Real-time PCR was performed in a 96-well format on a sequence detection system (LightCycler 480 II, Roche) with the following cycle conditions: 2 min at 95°C followed by 45 cycles of 30 s at 95°C, 30 s at 60°C and 45 s at 72°C, with a melting curve analysis at the end of the reaction.

Machine learning analyses

Decision Tree created a tree-like model of decisions and their possible consequences, allowing for easy interpretation and visualization of the model. Support Vector Machines (SVM) was particularly useful for dealing with these complex, non-linear data that could not be easily separated by a linear model. SVM tries to find the best hyperplane that separates the classes with the maximum margin. Random Forest is an ensemble learning method that combines multiple decision trees to improve model accuracy and prevent overfitting. It randomly selects a subset of features and observations for each tree, and then aggregates the results to make a final prediction. Partial Least Squares (PLS) is a supervised learning algorithm that can be used for regression and dimensionality reduction. It is particularly useful when dealing with high-dimensional data that contains many correlated variables. PLS identified the latent variables that capture the most important information in the data. ROC (Receiver Operating Characteristic) is a curve that

shows the performance of a binary classifier at different classification thresholds, while AUC (Area Under the Curve) is a single numerical value that summarizes the overall performance of the classifier.

Acknowledgments:

The NHLBI Recipient Epidemiology Donor Evaluation Study – IV – Pediatric (REDS-IV-P) domestic program is the responsibility of the following persons:

Hubs

A.E. Mast and L. Baumann Kreuziger, Versiti Wisconsin, Milwaukee, WI

E.A. Hod, Columbia University Medical Center, New York, NY and B.S. Sachais, New York Blood Center, New York, NY

B.S. Custer, Vitalant Research Institute, San Francisco, CA and E.P. Vichinsky, Benioff Children's Hospital Oakland, Oakland, CA

J.E. Hendrickson, Yale University School of Medicine, New Haven, CT and B.R. Spencer, American Red Cross, Dedham, MA

Data Coordinating Center

S.M. Mathew, Westat, Rockville, MD

N.L. Luban, Children's National Medical Center, Washington, D.C

Center for Laboratory Transfusion Science

P.J. Norris and M. Stone, Vitalant Research Institute, San Francisco, CA.

Publications Committee Chairman

P.M. Ness, Johns Hopkins University, Baltimore, MD

Steering Committee Chairpersons

C.D. Josephson, Emory University, Atlanta, GA

S.H. Kleinman, University of British Columbia, Victoria, BC, Canada

National Institute of Child Health and Human Development (NICHD)

R. Tamburro

National Heart, Lung, and Blood Institute, National Institutes of Health

M. Oullette, L. Welniak, and K. Malkin

Supplemental Table 1. Transfusion recipient demographic characteristics

Recipient Charactistic	
**Sex, N male (%)**	273 (55)
**Age, mean (SD)**	63 (16)
**Baseline BMI, mean (SD)**	28 (6.8)
**ABO Identical, N (%)**	456 (92)
**Race, N (%)**	
White	379 (76)
Black/African American	72 (14)
Asian	3 (0.6)
Other	4 (0.8)
Unknown	38 (7.7)
**Concomitant plasma Tx, N (%)**	14 (2.8)
**Concomitant platelet Tx, N (%)**	25 (5.0)
**Issue Location, N (%)**	
General Ward	234 (47)
ED	30 (6.0)
ProcSuite/OR	27 (5.4)
ICU	169 (34)
Outpatient	18 (3.6)
Other	8 (1.6)
Missing	10 (2.0)

BMI, body mass index; Tx, transfusion; ED, emergency department; ProcSuite, procedure suite; OR, operating room; ICU, intensive care unit

Supplemental Table 2. Specific HETEs associated with total EV counts

HETEs	rho	R^2	p-value
15(S)-HETE	0.357	0.127	0
17(S)-HDHA	0.32	0.102	0
5(S)-HETE	0.318	0.101	0
12(S)-HETE	0.3	0.09	0
12,13-DiHOME	0.247	0.061	0
8-iso-Prostaglandin E2	0.219	0.048	0
Prostaglandin E2	0.211	0.045	0
13-oxo-ODE	0.196	0.038	0
9-oxo-ODE	0.182	0.033	1.11E-15
Prostaglandin A2,B2	-0.142	0.02	5.77E-10
Leukotriene B4	0.132	0.017	8.07E-09
9(S)-HODE	-0.109	0.012	1.94E-06
13(S)-HODE	0.103	0.011	7.65E-06
9,10-DiHOME	0.101	0.01	1.14E-05
Glycochenodeoxycholic acid	0.094	0.009	4.27E-05
13,14-dihydro-15-keto-Prostaglandin E1, F2a	-0.089	0.008	0.00011
Glycoursodeoxycholic acid	0.084	0.007	0.00027
Cholic acid	0.078	0.006	0.00073
Taurochenodeoxycholic acid	0.067	0.005	0.0034
Glycodeoxycholic acid	0.066	0.004	0.0043
2,3-dinor-8-iso-Prostaglandin F2α	0.063	0.004	0.0058
Tauroursodeoxycholic acid	0.054	0.003	0.019
Taurodeoxycholic acid	0.052	0.003	0.024

Figure S1

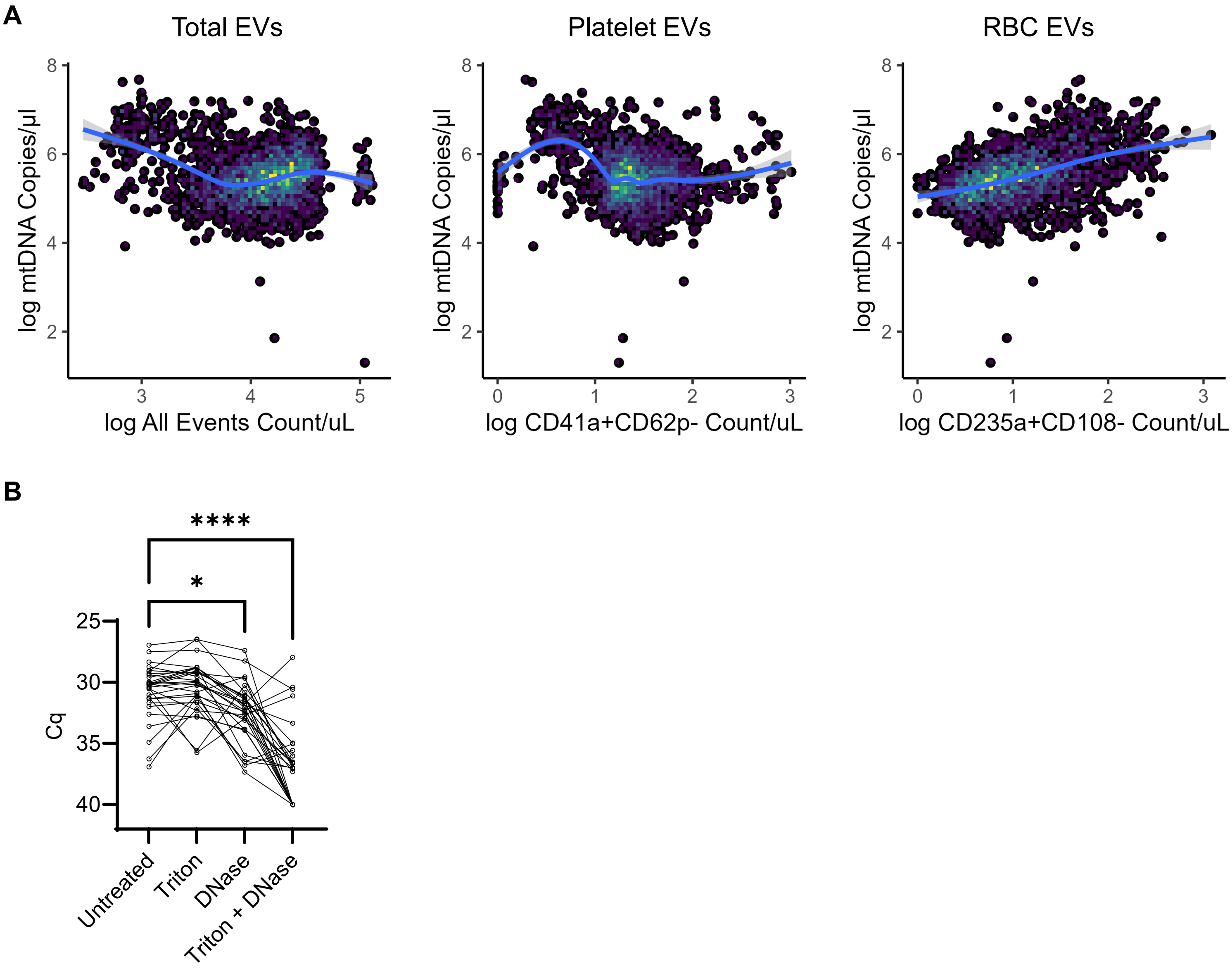

Supplemental Figure 1. Relationship between EVs and mtDNA levels. (A) EV and mtDNA from all three storage days were pooled and correlated by Spearman correlation. Dot plots show individual data measurements for mtDNA vs. the noted EV phenotype (n=651). The fitted line indicates the predicted Loess mean storage hemolysis for corresponding EV type, and the shaded areas highlight the 95% confidence intervals for the standard error of the mean. P-values are derived for Spearman correlation. (B) 30 RBC supernatant samples that were previously unthawed were tested by RT-PCR either untreated or after addition of the detergent Triton-X100, DNase, or both. Cq = quantification cycle, the point at which specific signal is detected. Undetected samples were assigned Cq=40. *p<0.05, ****p<0.0001

Figure S2

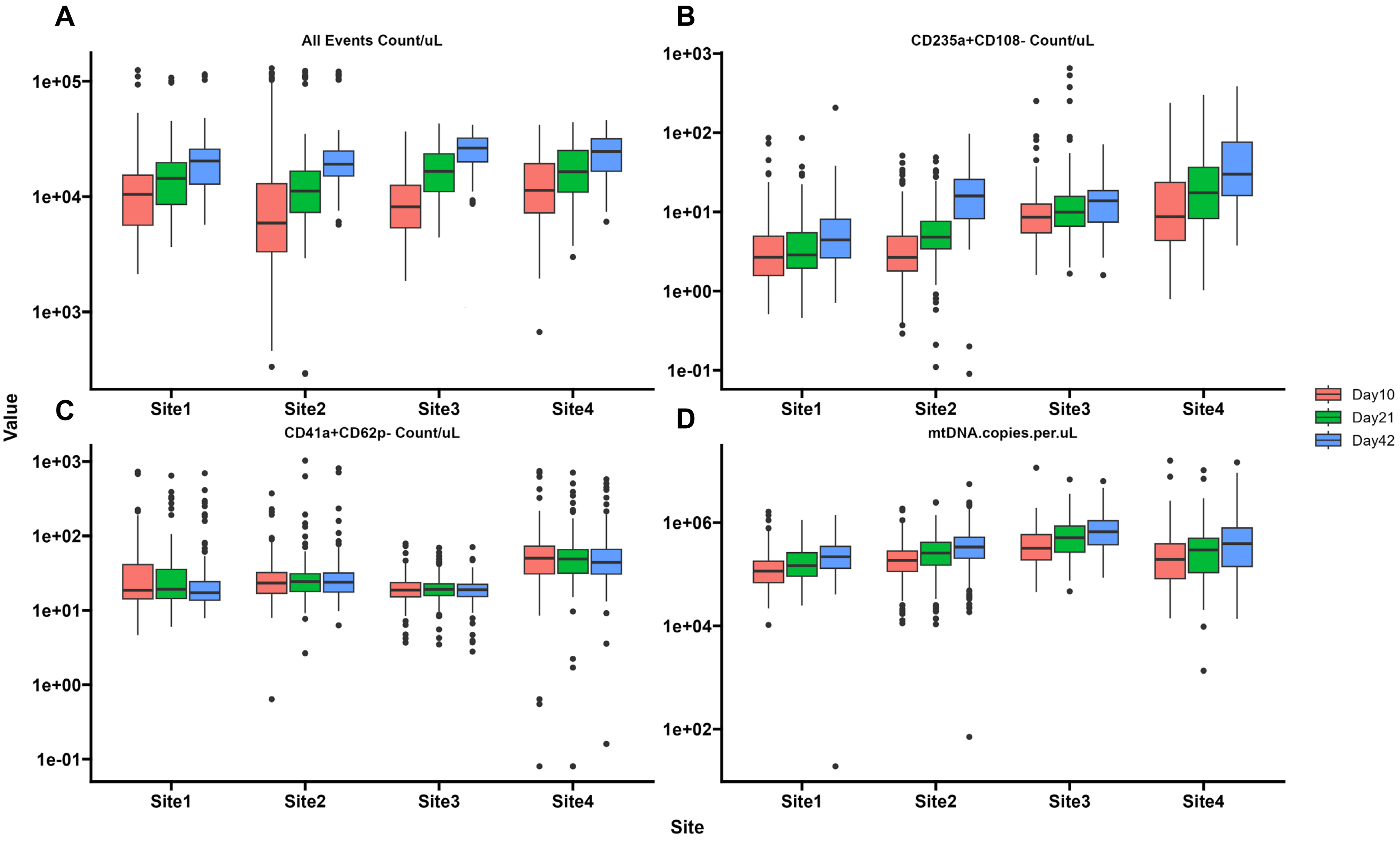

Supplemental Figure 2. Effect of delayed LR on EV and mtDNA levels. (A) Total EV count, (B) RBC EVs, (C) platelet EVs, and (D) mtDNA levels are shown at days 10, 21, and 42 of storage. Sites 1-3 performed immediate LR after collection, while site 4 delayed LR by 48 to 72 hours. Sites were compared by Wilcoxon test, and site 4 differed from the other three for RBC and platelet Evs ($p<0.0001$).

Figure S3

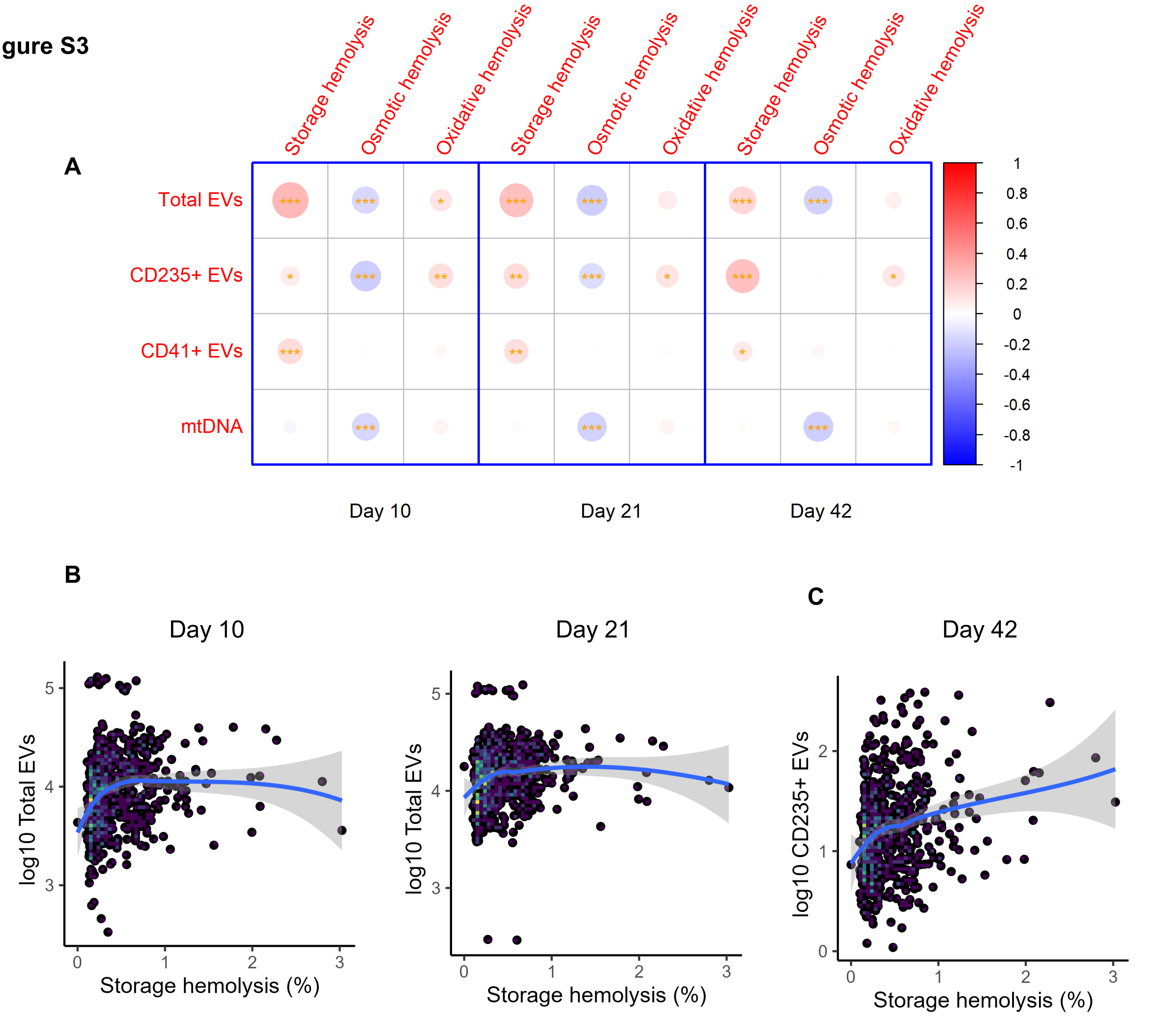

Supplemental Figure 3. Correlation between EV and mtDNA levels and hemolysis parameters. (A) EV and mtDNA levels were measured at days 10, 21, and 42 and correlated with the outcomes of storage, osmotic, and oxidative hemolysis measured at day 42 of RBC unit storage (n=651). The color of each shaded circle corresponds to the strength of the correlation (R), with positive correlations in red and negative in blue. Spearman *p<0.05, **p<0.01, ***p<0.001, ****p<0.0001. (B) Dot plots show individual data measurements for storage hemolysis vs. day 10 and 21 total EVs and (C) vs. day 42 RBC-derived EVs. The fitted line indicates the predicted mean storage hemolysis for corresponding EV type, and the shaded areas highlight the 95% confidence intervals for the standard error of the mean. The three dot plots all have significant positive correlations (Spearman, p<0.001).

Figure S4

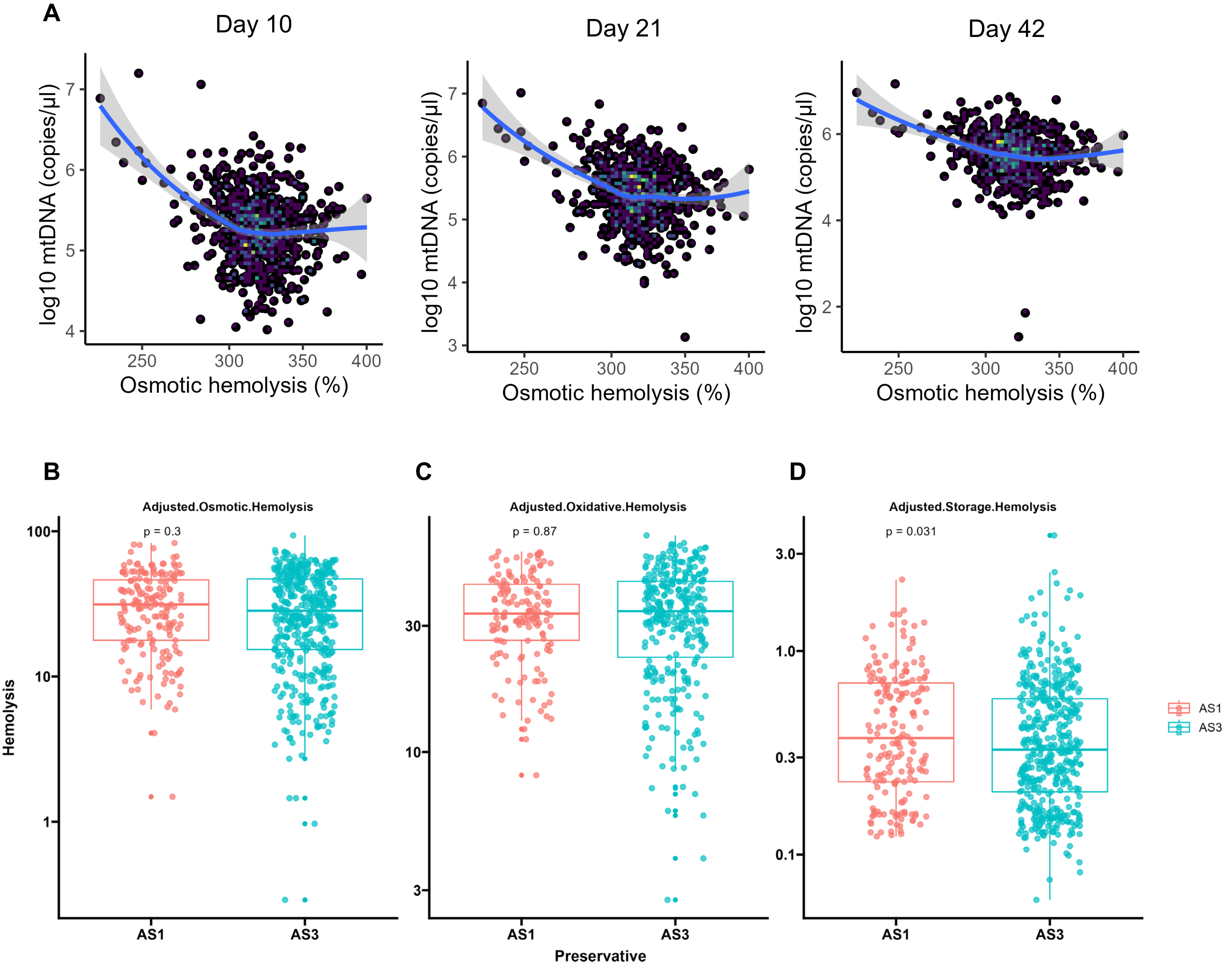

Supplemental Figure 4. Correlation between mtDNA levels and osmotic hemolysis. (A) Plots show the relationship between mtDNA and osmotic hemolysis at 10, 21, and 42 days of storage (n=651). The fitted line indicates the predicted mean osmotic hemolysis for corresponding mtDNA, and the shaded areas highlight the 95% confidence intervals for the standard error of the mean. The three dot plots all have significant negative correlations (Spearman, $p<0.001$). (B) End of storage osmotic hemolysis and (C) oxidative hemolysis did not differ by storage solution, while (D) storage hemolysis showed a significant difference ($p=0.031$, Wilcoxon text).

Figure S5

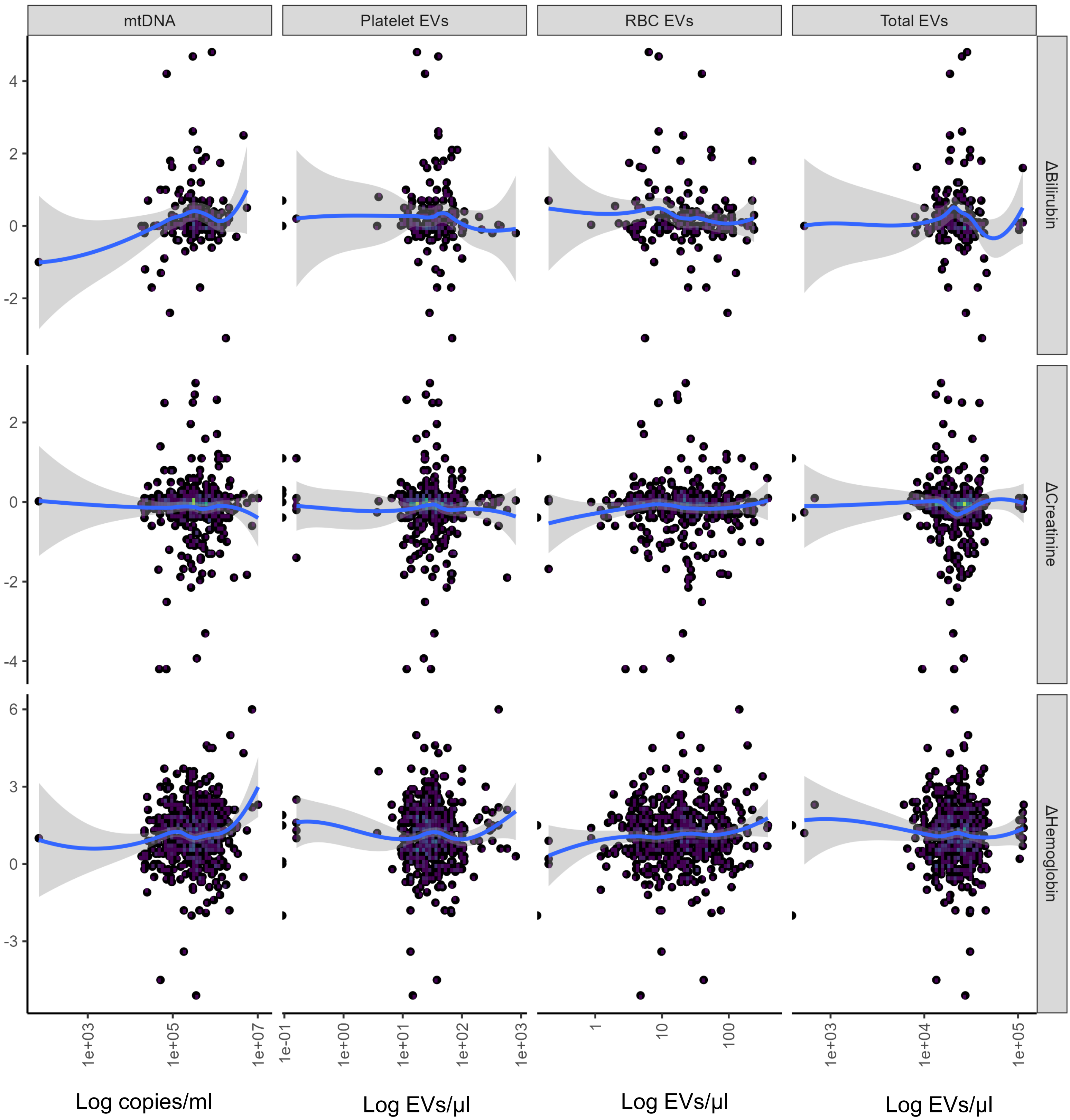

Supplemental Figure 5. Lack of correlation between mtDNA and EV phenotypes with change in bilirubin, creatinine, or hemoglobin after transfusion. The relationship between mtDNA, platelet EVs, RBC EVs, and Total EVs with values in transfused patients is shown. The top row shows data for change in total bilirubin pre- and post-transfusion (N=156). The middle row shows change in creatinine (N=414), and the bottom row shows change in hemoglobin (N=492). Both axes are plotted on a log scale. The fitted line indicates the predicted mean lab value for corresponding mtDNA or EV population, and the shaded areas highlight the 95% confidence intervals for the standard error of the mean. Values of each clinical parameter with pre-transfusion levels greater than the mean plus two standard deviations of the variable (1.5 for creatinine and 2.5 for total bilirubin) and were excluded from the correlation analysis between EV or mtDNA levels and the lab outcome. None of the relationships showed a significant Spearman correlation.

Figure S6

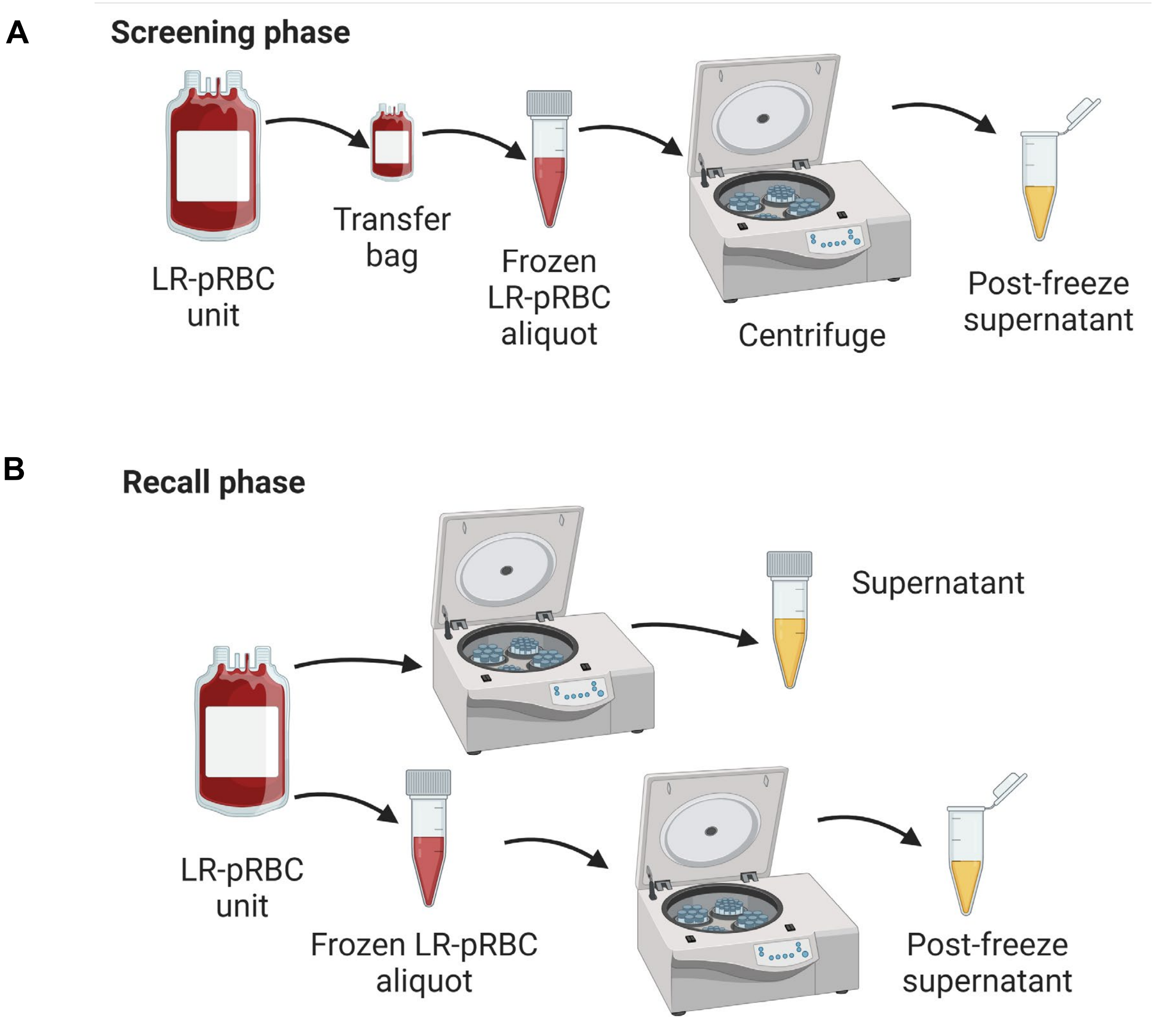

Supplemental Figure 6. Study design schematic. (A) In the screening phase of 13,403 blood donations, an aliquot of LR-pRBC was transferred to a transfer bag and stored for 42 days, at which point aliquots were snap-frozen and preserved at -80°C. For this study aliquots were thawed and centrifuged to generate a post-freeze supernatant. (B) In the recall phase 651 donors were selected from the parent study, and a unit of blood was aged for 42 days, with supernatant and LR-pRBC aliquots removed at 10, 21, and 42 days and cryopreserved at -80°C. For this study post-freeze supernatants were generated from a subset of samples for comparison to the screening samples (n=100).

Figure S7

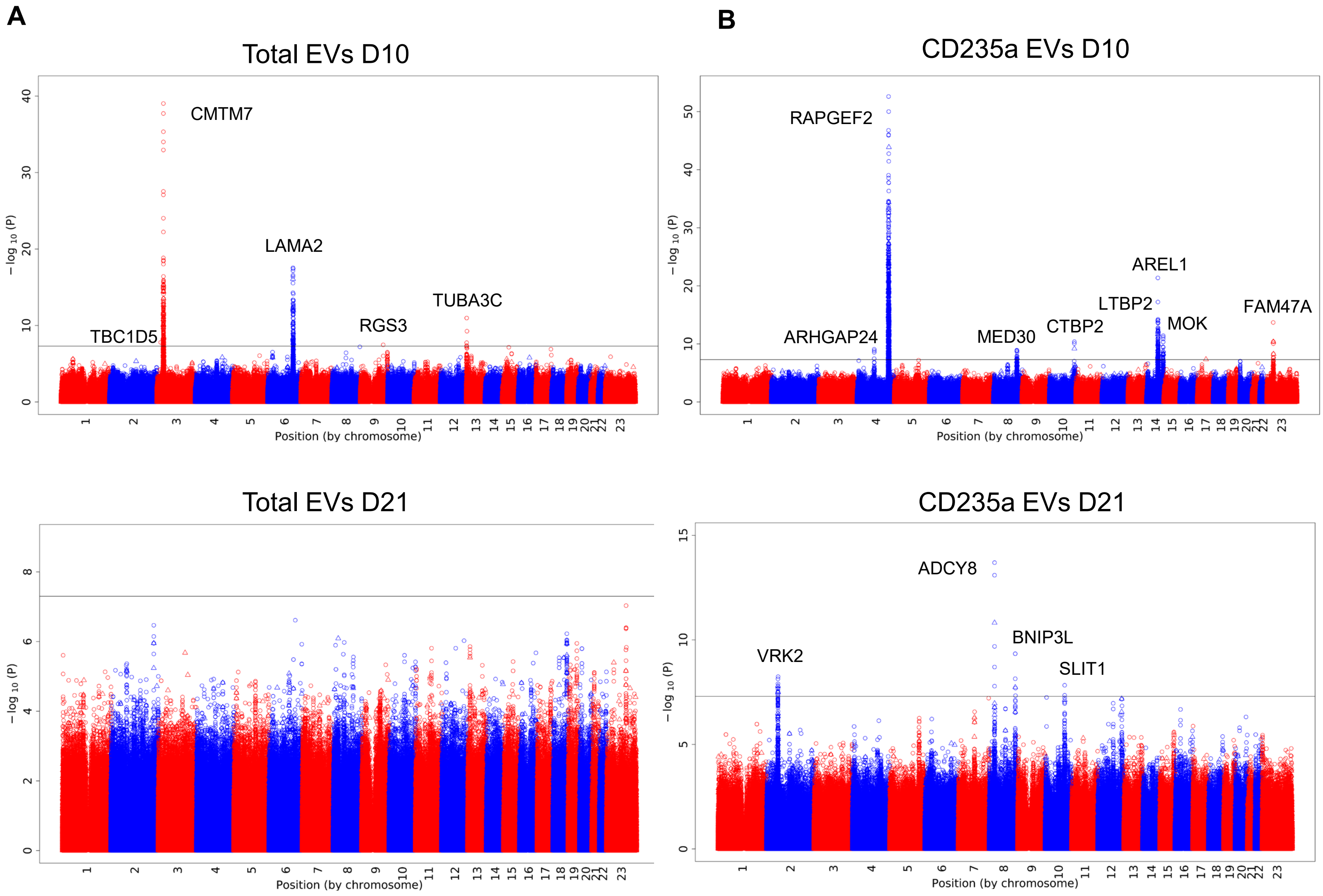
A
Total EVs D10
CMTM7
LAMA2
TUBA3C
RGS3
TBC1D5
− log 10 (P)
Position (by chromosome)
B
CD235a EVs D10
RAPGEF2
AREL1
LTBP2
MOK
FAM47A
CTBP2
MED30
ARHGAP24
Total EVs D21
CD235a EVs D21
ADCY8
BNIP3L
SLIT1
VRK2

Figure S7

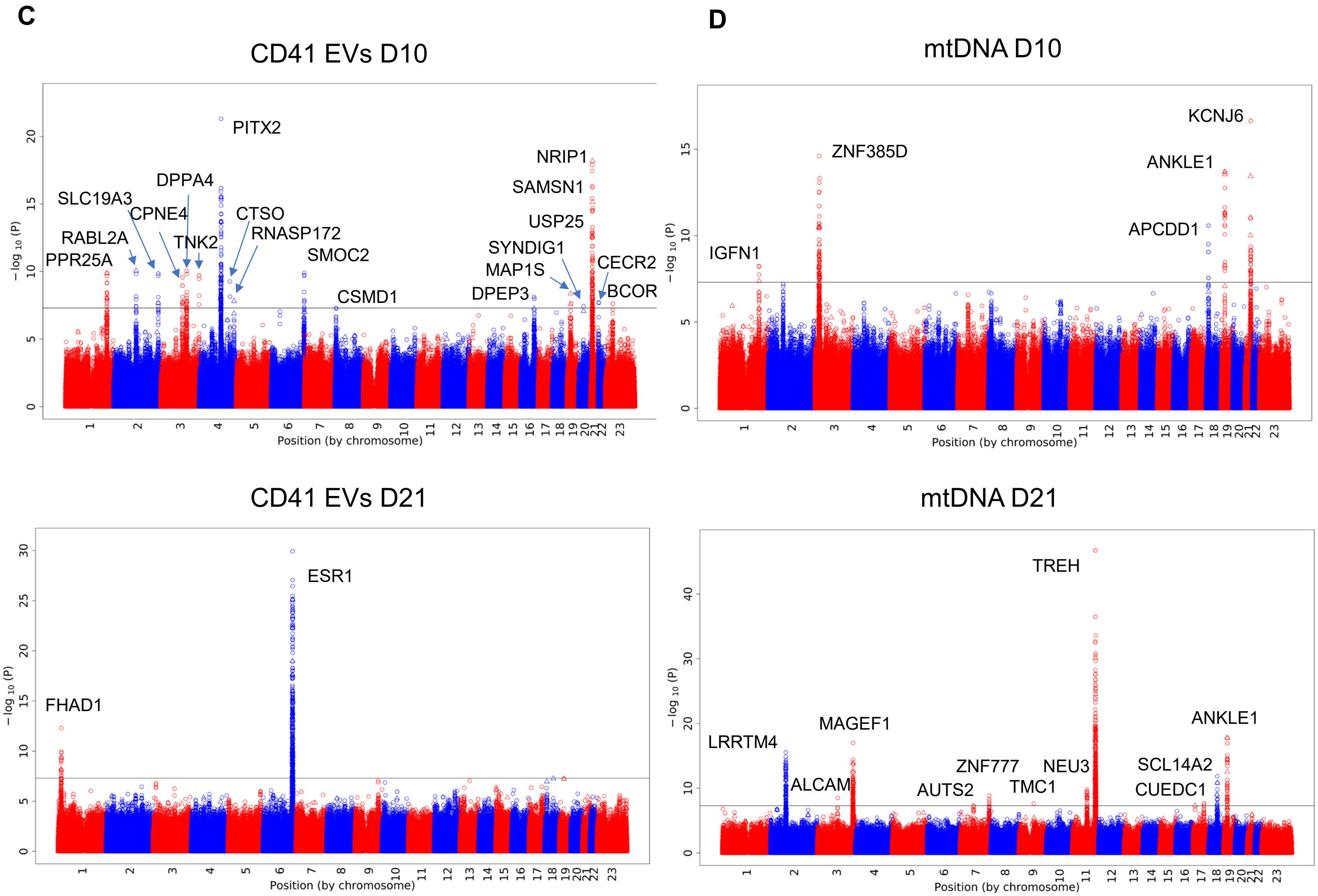

Supplemental Figure 7. Genetic polymorphisms associated with RBC unit EV and mtDNA levels at days 10 and 21 of storage. A QTL analysis was performed to associate SNPs with (A) total EVs, (B) CD235+ EVs, and (C) CD41a+ EVs, as well as (D) mtDNA in samples collected from 651 RBC units at days 10 and 21 of RBC unit storage. Associations with p value $<5x10^{-8}$ are labeled with the gene name, and the horizontal line on each plot represents $p=5x10^{-8}$.
